# Patterns in Cortical Connectivity for Determining Outcomes in Hand Function after Subcortical Stroke

**DOI:** 10.1371/journal.pone.0052727

**Published:** 2012-12-20

**Authors:** Dazhi Yin, Fan Song, Dongrong Xu, Bradley S. Peterson, Limin Sun, Weiwei Men, Xu Yan, Mingxia Fan

**Affiliations:** 1 Shanghai Key Laboratory of Magnetic Resonance, Key Laboratory of Brain Function Genomics, East China Normal University, Shanghai, China; 2 Department of Rehabilitation Medicine, Huashan Hospital, Fudan University, Shanghai, China; 3 MRI Unit, Department of Psychiatry, New York State Psychiatric Institute and Columbia University, New York, New York, United States of America; Hangzhou Normal University, China

## Abstract

**Background and Purpose:**

Previous studies have noted changes in resting-state functional connectivity during motor recovery following stroke. However, these studies always uncover various patterns of motor recovery. Moreover, subgroups of stroke patients with different outcomes in hand function have rarely been studied.

**Materials and Methods:**

We selected 24 patients who had a subcortical stroke in the left motor pathway and displayed only motor deficits. The patients were divided into two subgroups: completely paralyzed hands (CPH) (12 patients) and partially paralyzed hands (PPH) (12 patients). Twenty-four healthy controls (HC) were also recruited. We performed functional connectivity analysis in both the ipsilesional and contralesional primary motor cortex (M1) to explore the differences in the patterns between each pair of the three diagnostic groups.

**Results:**

Compared with the HC, the PPH group displays reduced connectivity of both the ipsilesional and contralesional M1 with bilateral prefrontal gyrus and contralesional cerebellum posterior lobe. The connectivity of both the ipsilesional and contralesional M1 with contralateral primary sensorimotor cortex was reduced in the CPH group. Additionally, the connectivity of the ipsilesional M1 with contralesional postcentral gyrus, superior parietal lobule and ipsilesional inferior parietal lobule was reduced in the CPH group compared with the PPH group. Moreover, the connectivity of these regions was positively correlated with the Fugl-Meyer Assessment scores (hand+wrist) across all stroke patients.

**Conclusions:**

Patterns in cortical connectivity may serve as a potential biomarker for the neural substratum associated with outcomes in hand function after subcortical stroke.

## Introduction

Strokes are a prevalent cause of adult-onset disability. Although a full recovery may occur after a stroke, more than 50% of the patients are left with residual motor deficits [1]. Particularly, deficits in hand function have a severe impact on the quality of life for stroke patients. Previous studies have consistently indicated that cerebral reorganization underlies motor recovery after stroke [2], [3], [4], [5], [6], [7], [8], [9], [10], [11]. However, various patterns of cerebral reorganization that contribute to motor recovery after a stroke have been reported using task-based, functional neuroimaging. For instance, shifts of primary sensorimotor cortex (S1M1) activation toward the infarct rim have been reported in patients with cortical infarcts, [3], [12] in regard to subcortical infarcts, both Pineiro et al [13] and Calautti et al [14] observed a posterior shift of S1M1 activation; Marshall et al [4] described an increase in the ratio of ipsilesional to the contralesional S1M1 activation, whereas Calautti et al [15] noted a decrease in activation of non-primary motor regions largely in the affected hemisphere; Ward et al [11] demonstrated that task-related activation decreased over sessions as a function of recovery in a number of primary and non-primary motor regions, however, Feydy et al [16] suggested that ipsilateral recruitment after stroke will persist if primary motor cortex (M1) is lesioned, otherwise, it will be transient. The disparity of the patterns of task-related activation might be attributed to the heterogeneity of the samples (e.g., variances in age, side of hemispheres, location and extent of lesion), degree of motor deficit at the time of imaging, differences in task paradigms, time from stroke onset, and synkinesia (mirror movement) [17]. Therefore, many factors that are not well-controlled may confound investigators in identifying the pattern of cerebral reorganization underlying motor recovery after a stroke. Furthermore, it is difficult to investigate severe hand function disabilities by task-based functional neuroimaging.

Recently, increasing attention has been focused on resting-state functional magnetic resonance imaging (fMRI), which mainly investigates low-frequency (0.01 Hz–0.08 Hz) fluctuations (LFFs) of blood oxygenation level-dependent (BOLD) fMRI signals in the resting state. The data from resting-state fMRIs are considered to reflect spontaneous neuronal activities [18], [19], [20], [21]. Moreover, Bianciardi and colleagues [22] suggested that resting fluctuations of BOLD signals correspond to neuronal activation during task performance. This is an attractive technique for studying stroke patients because it is noninvasive and does not require patients to perform complicated tasks to probe spontaneous brain activity. In contrast to task-based fMRI, resting-state fMRI can effectively remove the confounding factors from the presence of synkinesia (mirror movement) and the variances induced by different task paradigms. Moreover, resting-state fMRI is apparently more suitable for studying the patients with severe disabilities of hand function. Indeed, resting-state functional connectivity was principally used to explore interregional temporal correlations of low-frequency fluctuations in BOLD signals. Regions for which BOLD signal fluctuations exhibit a high degree of temporal correlation are presumed to constitute a specific functional network [23], [24], [25]. Resting-state functional connectivity routinely reflects functional integrity and was used to detect dysfunctions in brain networks, including Alzheimer's disease, [26], [27] schizophrenia, [28] depression, [29] and attention deficit hyperactivity disorder [30].

In stroke patients, a resting-state network of motor execution has been used to investigate the dynamic functional reorganization following motor recovery, which revealed increased functional connectivity of ipsilesional M1 with contralesional S1M1 and premotor cortex during the motor recovery [31]. Also, in acute stroke patients, disruptions in the attention and somatomotor networks were used to predict post-stroke performance [32]. In addition, longitudinal changes in resting-state functional connectivity of the ipsilesional M1 with contralesional S1M1, frontal cortex, parietal cortex and cerebellum during motor recovery after stroke have been recently reported [33]. However, the results of these studies encompass various patterns of motor recovery. Therefore, recruiting subgroups of stroke patients with different outcomes in motor function and carefully controlling sample homogeneity appears to be required for the successful interpretation of different motor recovery patterns. Few studies have explored whether different outcomes in hand function can be attributed to the differences in connectivity of the ipsilesional M1 with some motor-related regions.

In the clinic, many stroke patients that appear to have similar locations and extent of lesions may actually have extremely different outcomes in hand function. Some have regained certain practical abilities in hand function after the stroke, whereas others have lost all functional capacity of their hand. The former are regarded as having partially paralyzed hands (PPH) and the latter as having completely paralyzed hands (CPH). Previous longitudinal studies suggested that changes in the pattern of motor-related brain activation do not occur as a function of time after stroke, but rather as a function of motor recovery [11]. However, how functional reorganization contributes to differences in outcome in hand functioning has not been completely identified.

In our current study, we performed resting-state, functional connectivity analysis of the ipsilesional M1 to investigate subgroups of stroke patients with different outcomes in hand function. We hypothesized that the PPH and CPH groups would exhibit differences in connectivity of the ipsilesional M1 with contralesional S1M1, frontal cortex, parietal cortex or cerebellum compared to healthy controls (HC). Moreover, we hypothesized that the differences of the connectivity between the CPH and PPH groups would correlate with scores on the Fugl-Meyer Assessment (FMA) (hand+wrist) across all stroke patients. In addition, whether the infarcts may disrupt the connectivity of contralesional M1, which mainly dominates the movement of unaffected hand, has rarely been studied. Therefore, we were also interested in investigating the functional connectivity of contralesional M1 in the CPH and PPH groups, and compared them with HC.

There are three key aspects to our current study. First, the relative homogeneity of the samples was carefully controlled, which is helpful for reducing individual variability and partially increasing statistical power. Second, to our knowledge, this is the first study to employ resting-state, functional connectivity analysis to investigate subgroups of stroke patients with different outcomes in hand function. Third, we primarily focused on the reorganization of cortical connectivity, which should not be confused with the primary lesion in the subcortex.

## Materials and Methods

### Participants

From May 26, 2010 to November 28, 2011, 24 patients with pure motor deficits from a subcortical stroke that occurred in the left motor pathway were selected from both outpatient and inpatient services at the Huashan hospital affiliated with Fudan University. The patients were divided into two subgroups: the PPH group (12 patients, 11 males, age±SD: 61±7.8 (years)) and the CPH group (12 patients, 7 males, age±SD: 61±7.2 (years)). Twenty-four HC (14 males, age±SD: 62±9.8 (years)), matched for both age and handedness, were recruited from local communities. Both the Mini-Mental State Examination (MMSE) and FMA were administered to all stroke patients. Inclusion criteria were as follows: (1) all patients were first-onset stroke victims; (2) all participants were right handed; (3) all participants had sufficient cognitive abilities (MMSE>24); (4) at least 3 months from stroke onset was allowed (6 months for CPH patients because we identified CPH with a prerequisite that is at least 6 months from stroke onset); and (5) age of the participants was between 45–80 years. Exclusion criteria were as follows: (1) participants with a contraindication to MRI; (2) patients with quadriplegia; (3) participants with a prior history of neurological and psychiatric disorders; (4) participants with diabetes; and (5) participants suffering from previous hand dysfunctions. Patient clinical characteristics and demographic data are summarized in Table 1, and the lesion with the maximum area in each stroke patient is shown in Figure S1. The protocol for this prospective study was approved by the Institutional Ethics Committee of East China Normal University, Shanghai, China, and all participants or their guardians signed informed consent forms.

**Table 1:** Clinical and demographic data of 24 subcortical stroke patients enrolled in this study.

Case	Gender	Age(years)	Location of lesion	Course of disease (months)	handed	MMSE	FMA Scores (hand+wrist)	
**PPH GROUP**								
01	M	56	L,IC,BG,Th	14	R	30	23	
02	M	60	L,IC,Th*	53	R	30	12	
03	F	48	L,IC,BG	23	R	29	6	
04	M	76	L,IC	21	R	27	22	
05	M	60	L,IC,BG	36	R	30	6	
06	M	71	L,IC,Th*	22	R	27	11	
07	M	63	L,IC,BG,Th	3	R	30	13	
08	M	54	L,IC,Th*	3	R	29	23	
09	M	60	L,BG,Th,*	11	R	30	23	
10	M	65	L,IC,Th	12	R	29	23	
11	M	53	L,IC,BG,Th*	22	R	26	15	
12	M	65	L,IC,BG*	6	R	29	20	
**CPH GROUP**								
01	M	62	L,BG,IC,Th	6	R	28	4	
02	M	56	L,IC	7	R	27	6	
03	M	56	L,BG,IC,Th*	21	R	27	1	
04	M	57	L,IC,Th	19	R	28	0	
05	F	75	L,IC,CR	24	R	29	4	
06	M	63	L,BG,IC,Th*	16	R	28	1	
07	F	65	L,IC,Th	17	R	30	1	
08	F	68	L,BG,IC,Th*	62	R	28	0	
09	M	68	L,IC,Th*	47	R	29	1	
10	M	53	L,BG,IC,Th*	86	R	30	1	
11	F	50	L,BG,IC,Th*	13	R	28	0	
12	M	61	L,IC,BG	6	R	29	4	

Note: M = male; F = female; L = left; R = right; BG = basal ganglia; IC = internal capsule; Th = thalamus; CR = coronal radiate. * indicate the character of the lesion is hemorrhage, others are ischemia.

We identified CPH and PPH using Paralyzed Hand Function Assessment, which involves five practical actions of hand in daily life (Figure S2). All the stroke patients were evaluated using this assessment. All those who could not complete any action were regarded as CPH, and those who could complete at least one of the five actions were regarded as PPH. The assessment was performed by two experienced doctors from the Department of Rehabilitation Medicine, Huashan hospital.

### Data Acquisition

All images were acquired using a Siemens Trio 3.0 Tesla MRI scanner (Siemens, Erlangen, Germany) at the Shanghai Key Laboratory of Magnetic Resonance, East China Normal University. The head of each participant was snugly fixed using foam pads to reduce both head movements and scanner noise. Whole-brain, resting-state fMRI data were acquired using an echo-planar imaging (EPI) sequence: 30 axial slices, thickness  = 4 mm, gap  = 0.8 mm, matrix  = 64×64, repetition time  = 2000 ms, echo time  = 30 ms, flip angle  = 90° and a field of view  = 220 mm×220 mm. Three-dimensional (3D) structural images of T1-weighted images covering the entire brain were obtained in a sagittal orientation by employing magnetization prepared by rapid gradient echo sequence (MPRAGE): 192 slices per slab, thickness  = 1 mm, gap  = 0.5 mm, repetition time  = 1900 ms, echo time  = 3.42 ms, field of view  = 240 mm×240 mm and a matrix  = 256×256. To identify the location of the lesion, T2-weighted images were collected using a turbo-spin-echo sequence: 30 axial slices, thickness  = 5 mm, no gap, repetition time  = 6000 ms, echo time  = 93 ms, field of view  = 220 mm×220 mm and a matrix  = 320×320. During the acquisition of the EPI data, the participants were instructed to remain awake, remain motionless, and to relax with their eyes closed and try not to think about anything in particular. Each scan lasted for 8 minutes and 6 s; the first 6 s was consumed on the dummy scan. Thus, we collected a total of 240 image volumes.

### Preprocessing of the Functional MRI Data

Preprocessing of the fMRI data was performed using Statistical Parametric Mapping (SPM8, http://www.fil.ion.ucl.ac.uk/spm). We discarded the first 10 volumes of the dataset of each participant to allow for magnetization equilibrium, thus leaving 230 volumes for further analysis. The images were corrected for the delay in slice acquisition and were co-registered to the first image to correct for rigid-body head movement. Excessive motion was defined as more than 2.5 mm of translation or greater than a 2.5 degree rotation in any direction. One stroke patient was excluded due to excessive motion. Subsequently, each individual, T1-weighted, 3D structural image was co-registered to the mean functional image after motion correction using linear transformation. The transformed structural images were then segmented into gray matter (GM), white matter (WM) and cerebrospinal fluid (CSF) using a unified segmentation algorithm [34]. The functional images underwent motion correction and were spatially normalized to the Montreal Neurological Institute (MNI) space and then resampled to a 3 mm isotropic voxel using the normalization parameters estimated during unified segmentation. Finally, the normalized images were spatially smoothed using an isotropic Gaussian filter at full width at a half maximum (FWHM) of 4 mm.

### Analysis of Functional Connectivity

**Figure 1:**
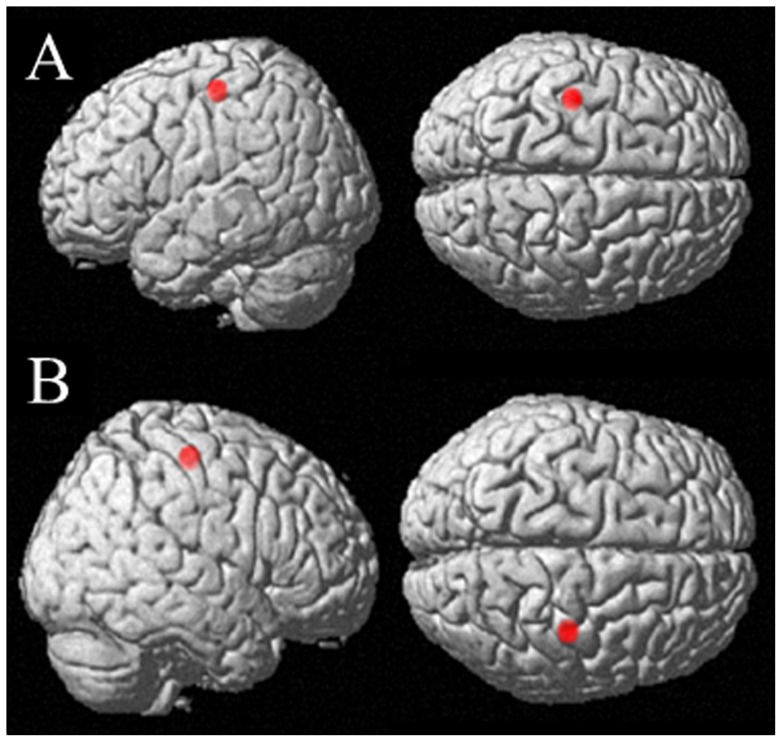
Seed regions of interest (ROIs) (red) for functional connectivity. A: Ipsilesional M1, MNI coordinate = −38, −22, 56, radius = 6 mm; B: Contralesional M1, MNI coordinate = 38, −22, 56, radius = 6 mm.

Following preprocessing, the smoothed images were further processed using the software Resting-State fMRI Data Analysis Toolkit (REST, http://restfmri.net) [35]. First, we removed the linear trend and the temporal band-pass (0.01 Hz–0.08 Hz) filtering to reduce low-frequency drift and high-frequency respiratory and cardiac noise. Subsequently, we selected the seed ROI (region of interest) in the ipsilesional M1 (left side in our study) at the Montreal Neurological Institute (MNI) coordinate of −38, −22, 56, which locates on the hand representation of M1 [31]. The radius of 6 mm was used to define the spatial extent of the ROI. We then used the seed ROI to perform functional connectivity analysis in the three diagnostic groups (Figure 1).

We were also interested in exploring what patterns of connectivity of the contralesional M1 may appear in subgroups of stroke patients with different outcomes in hand function compared with HC, which has rarely been investigated. Therefore, the seed ROI for functional connectivity in the contralesional M1 (right side in our study) was selected at the MNI coordinate of 38, −22, 56, which locates on the hand representation of M1, [31] and a radius of 6 mm was used to define the spatial extent of the ROI. (Figure 1).

The functional connectivity of each individual participant was calculated as follows. First, three nuisance covariates (i.e., an fMRI time series representing the global trend for the entire brain, WM and CSF) were obtained. Second, the reference time course was calculated by averaging the time series of all voxels in the seed ROI. Third, a Pearson's correlation analysis was performed based on the voxel between the reference time course and time series of each voxel in the brain using the global signal, WM signal, CSF signal and the 6 parameters of head motion as nuisance covariates. Finally, the resulting correlation coefficients were transformed into z-scores using Fisher's z-transformation so that their distributions could better satisfy normality [36]. Subsequently, a two-tailed, two sample t-test was conducted on the z-maps in each pair of the three diagnostic groups. Multiple comparisons correction was performed using AlphaSim program in AFNI (see AlphaSim in AFNI, http://afni.nimh.nih.gov/pub/dist/doc/manual/AlphaSim.pdf) and a corrected threshold of p<0.05 was utilized, with a combined cutoff value of p<0.01 and a minimum cluster size of 486 mm^3^ (18 voxels). The statistically significant differences of functional connectivity between each pair of the three diagnostic groups were overlapped on the render views.

### Correlation Analysis between Index of Functional Connectivity and FMA Scores

To assess whether the differences in connectivity of the ipsilesional M1 between the CPH and PPH groups varies with hand motor deficits, a further correlation analysis between the index of functional connectivity and FMA scores (hand+wrist) was performed. All of the three regions that showed significant differences in connectivity of the ipsilesional M1 between the CPH and PPH groups were examined, including contralesional postcentral gyrus, superior parietal lobule and ipsilesional inferior parietal lobule (IPL). First, the mean of functional connectivity of the ipsilesional M1 across all voxels of the identified cluster was extracted from each individual stroke patient. Subsequently, a non-parametric Spearman correlation was performed between the index of functional connectivity and FMA scores (hand+wrist) across all stroke patients. A p-value <0.05 (uncorrected) was considered to be statistically significant.

## Results

### Connectivity of the Ipsilesional M1 between Each Pair of the Three Diagnostic Groups

**Figure 2:**
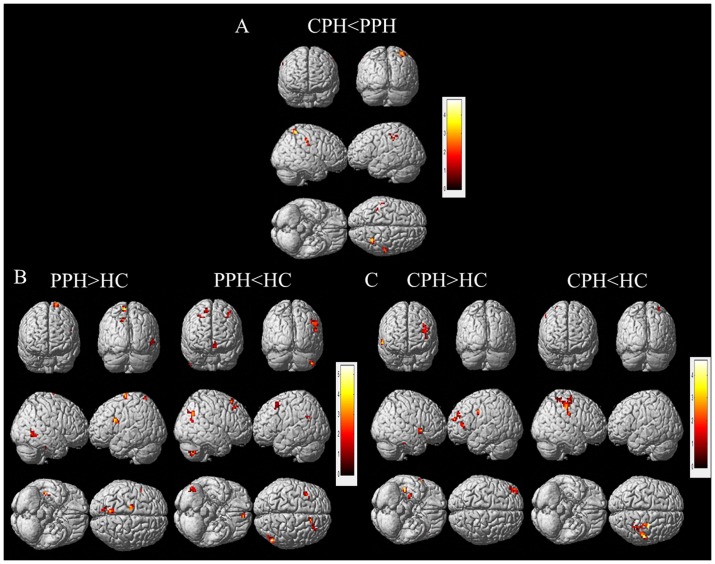
Significant differences in the functional connectivity of ipsilesional M1 between each pair of the three diagnostic groups. A: CPH versus PPH; B: PPH versus HC; C: CPH versus HC. The threshold was set at a combined cutoff value of p<0.01 and a minimum cluster size of 486 mm^3^ (18 voxels) to yield a corrected threshold of p<0.05 (AlphaSim corrected). Clusters with significant differences were overlapped on render views (posterior-anterior (row 1), right-left (row 2), inferior-superior (row 3)). Color scale = t values.

Compared with the HC group, the PPH patients displayed reduced connectivity of the ipsilesional M1 with bilateral prefrontal gyrus and contralesional cerebellum posterior lobe. In contrast, connectivity with contralesional S1M1 was reduced in the CPH group. Additionally, we observed increased connectivity of the ipsilesional M1 with regions of the ipsilesional premotor cortex, postcentral gyrus, inferior frontal gyrus and contralesional middle temporal gyrus in the PPH group compared with the HC group. There was increased connectivity with ipsilesional precentral gyrus, middle frontal gyrus, inferior frontal gyrus and contralesional superior temporal gyrus in CPH patients compared with the HC group. Furthermore, connectivity of the ipsilesional M1 with contralesional postcentral gyrus, superior parietal lobule and ipsilesional IPL were reduced in the CPH group compared with the PPH group. And, no regions were observed showing increased connectivity with ipsilesional M1 in the CPH group compared with PPH group. (Figure 2 and Table 2).

**Table 2:** The significant differences of functional connectivity of the ipsilesional M1 between each pair of the three diagnostic groups.

Between-group Comparison and Regions	MNI Coordinates			Peak t-Score	Number of Voxels	Volume (mm3)
	x	y	z			
**CPH<PPH**						
Inferior Parietal Lobule (IL)	−39	−39	45	3.69	29	783
Postcentral Gyrus (CL)	54	−24	45	3.28	28	756
Superior Parietal Lobule (CL)	36	−51	63	4.82	32	864
**PPH>HC**						
Premotor cortex (IL)	−18	−12	78	4.72	28	756
Postcentral Gyrus (IL)	−9	−57	69	5.43	13	351
Precuneus (IL)	−12	−72	45	3.87	21	567
Inferior Frontal Gyrus (IL)	−42	12	27	4.64	21	567
Middle Temporal Gyrus (CL)	48	−51	−3	3.95	24	648
**PPH<HC**						
Medial Frontal Gyrus (IL)	−6	51	−6	4.16	20	540
Precuneus(IL)	−9	−51	30	3.87	41	1107
Angular Gyrus(IL)	−42	−54	21	3.35	20	540
Middle Frontal Gyrus (IL)	−39	18	57	3.66	21	567
Middle Frontal Gyrus (CL)	30	33	48	3.29	21	567
Superior Frontal Gyrus (CL)	12	24	60	3.30	17	459
Angular Gyrus (CL)	54	−66	21	3.68	35	945
Cerebellum Posterior Lobe (CL)	45	−60	−42	4.18	27	729
**CPH>HC**						
Precentral Gyrus (IL)	−48	−3	36	4.01	23	621
Middle Frontal Gyrus (IL)	−42	54	21	4.16	28	756
Inferior Frontal Gyrus (IL)	−45	33	6	3.78	21	567
Superior Temporal Gyrus (CL)	63	12	0	4.54	15	405
**CPH<HC**						
Precentral Gyrus (CL)	27	−21	57	4.28	55	1485
Postcentral Gyrus(CL)	51	−24	48	4.00	91	2457

Note: Between-group analysis was performed using a two-tailed two sample t-test. The threshold was set at a combined cutoff value of p<0.01 and a minimum cluster size of 486 mm^3^ (18 voxels), yielding a corrected threshold of p<0.05 (AlphaSim corrected). CPH: Completely Paralyzed Hands; PPH: Partially Paralyzed Hands; HC: Healthy Controls; IL: ipsilesional; CL: contralesional.

### Connectivity of the Contralesional M1 between Each Pair of the Three Diagnostic Groups

**Figure 3:**
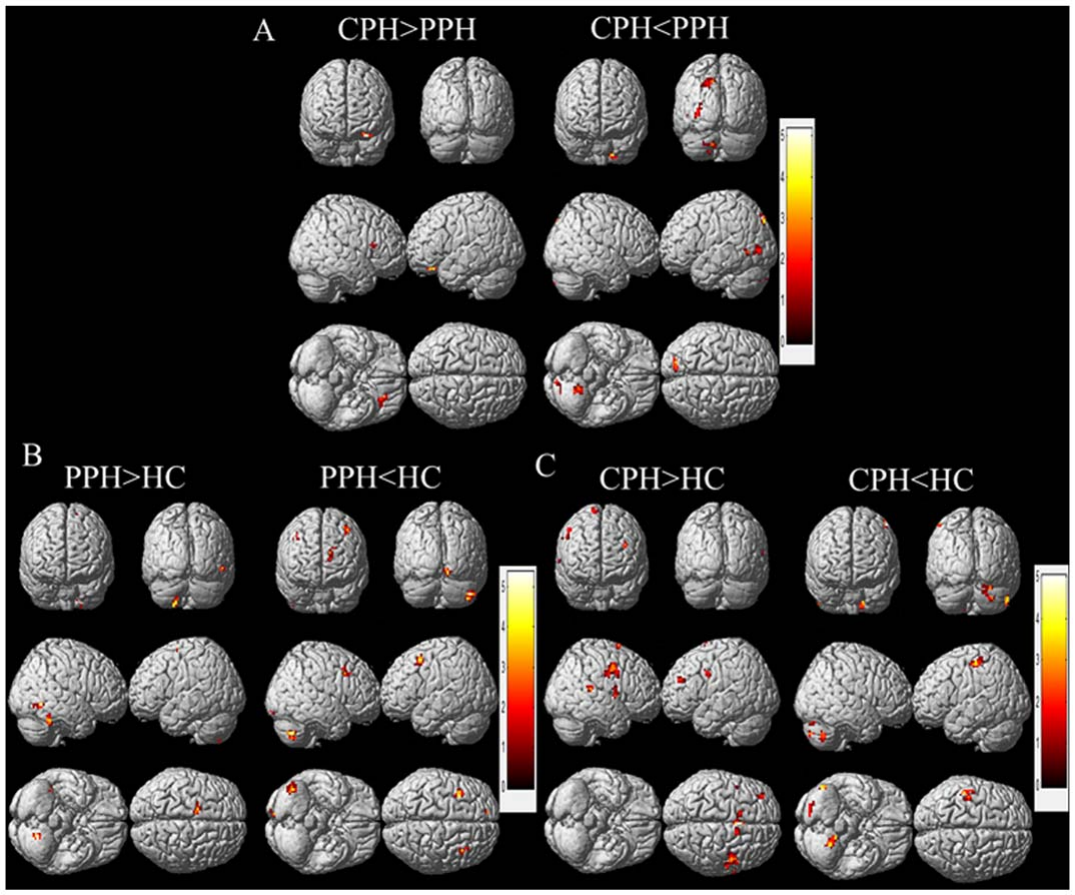
Significant differences in functional connectivity of contralesional M1 between each pair of the three diagnostic groups. A: CPH versus PPH; B: PPH versus HC; C: CPH versus HC. The threshold was set at a combined cutoff value of p<0.01 and a minimum cluster size of 486 mm^3^ (18 voxels) to yield a corrected threshold of p<0.05 (AlphaSim corrected). Clusters with significant differences were overlapped on render views (posterior-anterior (row 1), right-left (row 2), inferior-superior (row 3)). Color scale = t values.

Compared with the HC group, the PPH patients displayed reduced connectivity of the contralesional M1 with bilateral prefrontal gyrus and contralesional cerebellum posterior lobe. The connectivity with ipsilesional postcentral gyrus and bilateral cerebellum posterior lobe was reduced in the CPH group. Additionally, we observed increased connectivity in the contralesional M1 with regions of ipsilesional cerebellum posterior lobe and supplementary motor area (SMA) in the PPH group compared with the HC group. There was also increased connectivity with bilateral precentral gyrus, bilateral SMA, ipsilesional cingulate gyrus and middle frontal gyrus in the CPH patients compared to the HC group. In addition, we detected reduced connectivity of the contralesional M1 with ipsilesional cerebellum posterior lobe, temporal lobe and occipital lobe, and increased connectivity with ipsilesional orbitofrontal cortex in CPH patients compared with PPH patients (Figure 3 and Table 3).

**Table 3:** The significant differences of functional connectivity of the contralesional M1 between each pair of the three diagnostic groups.

Between-group Comparison and Regions	MNI Coordinate			Peak t-Score	Number of Voxels	Volume (mm3)
	X	y	z			
**CPH>PPH**						
Orbitofrontal cortex (IL)	−30	30	−21	4.34	14	378
**CPH<PPH**						
Cerebellum Posterior Lobe (IL)	−15	−54	−48	3.71	43	1161
Temporal Lobe (IL)	−36	−48	−3	4.06	28	756
Occipital Lobe (IL)	−9	−84	48	5.14	72	1944
**PPH>HC**						
Cerebellum Posterior Lobe (IL)	−21	−63	−57	3.88	17	459
Temporal Lobe (CL)	45	−45	−24	4.84	31	837
Supplementary Motor Area (IL)	−12	−6	60	3.93	11	297
**PPH<HC**						
Cerebellum Posterior Lobe (CL)	42	−60	−48	3.54	36	972
Middle Frontal Gyrus (IL)	−39	12	51	4.13	36	972
Medial Frontal Gyrus (IL)	−18	39	24	3.33	23	621
Middle Frontal Gyrus (CL)	42	15	39	3.59	20	540
Occipital Lobe (CL)	12	−87	−15	3.83	22	594
**CPH>HC**						
Precentral Gyrus (CL)	48	0	45	5.02	116	3132
Precentral Gyrus (IL)	−45	0	33	3.71	19	513
Cingulate Gyrus (IL)	0	9	42	4.87	124	3348
Supplementary Motor Area (IL)	−2	6	48	4.74	69	1863
Supplementary Motor Area (CL)	15	6	75	3.74	14	378
Temporal Gyrus (CL)	36	0	−15	4.72	44	1188
Middle Frontal Gyrus (IL)	−36	42	27	4.09	18	486
**CPH<HC**						
Postcentral Gyrus(IL)	−51	−30	57	3.51	39	1053
Cerebellum Posterior Lobe (IL)	−24	−51	−45	4.85	41	1107
Cerebellum Posterior Lobe (CL)	54	−66	−39	4.53	55	1485

Note: Between-group analysis was performed using a two-tailed two sample t-test. The threshold was set at a combined cutoff value of p<0.01 and a minimum cluster size of 486 mm^3^ (18 voxels), yielding a corrected threshold of p<0.05 (AlphaSim corrected). CPH: Completely Paralyzed Hands; PPH: Partially Paralyzed Hands; HC: Healthy Controls; IL: ipsilesional; CL: contralesional.

### Correlation between the Index of Functional Connectivity and FMA Scores

**Figure 4:**
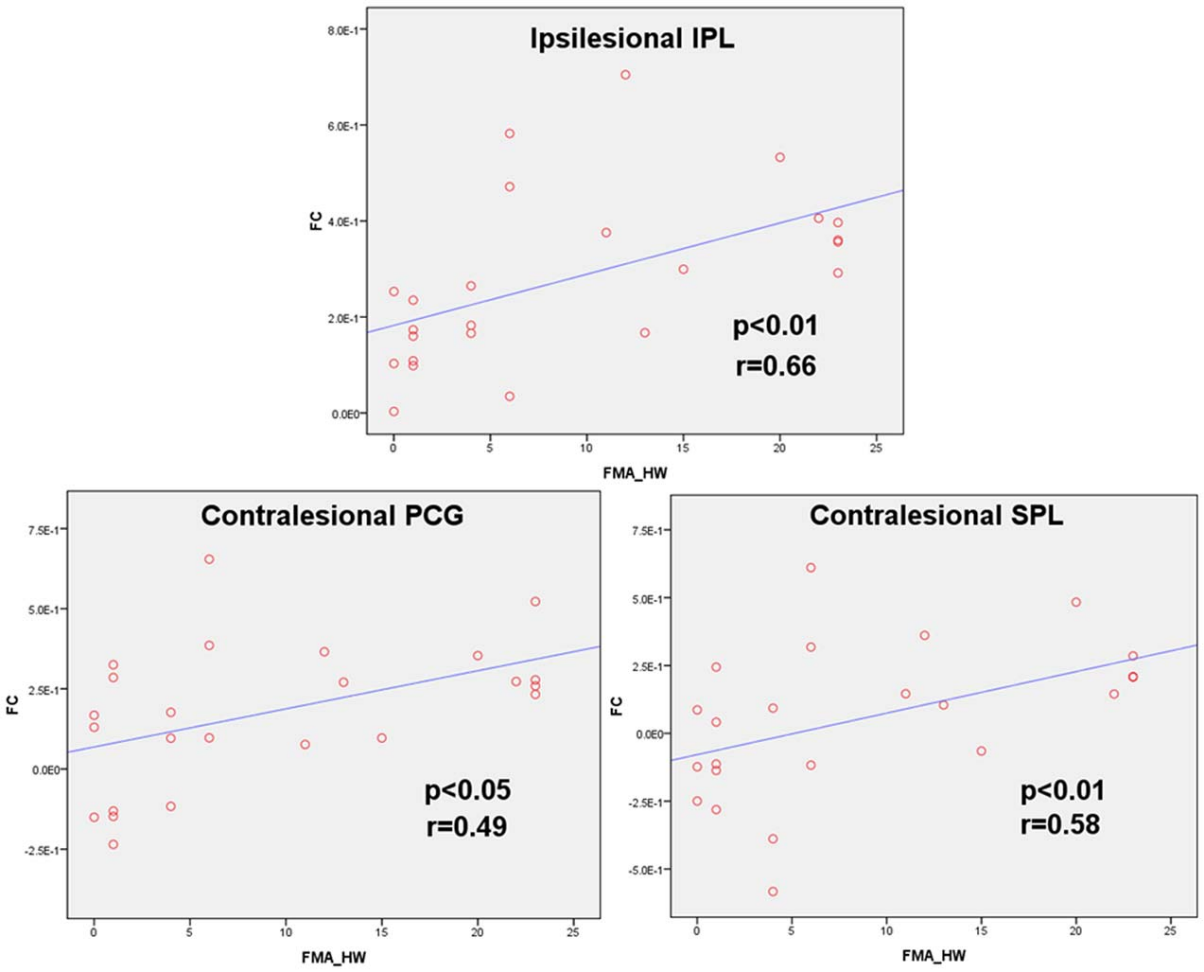
Functional connectivity of the ipsilesional M1 positive correlation with Fugl-Meyer Assessment (FMA) scores (hand+wrist). IPL: inferior parietal lobule; PCG: postcentral gyrus; SPL: superior parietal lobule; FC: functional connectivity; HW: hand+wrist.

Significant correlation between the index of functional connectivity and FMA scores (hand+wrist) in the regions identified as having significant differences in connectivity of the ipsilesional M1 between the CPH and PPH groups was revealed. Significant positive correlation was determined in the regions of ipsilesional IPL, contralesional postcentral gyrus and superior parietal lobule. The correlation coefficients are 0.66, 0.49, and 0.58, respectively (Figure 4). Both regions showing greater and lesser connectivity in CPH than PPH were examined and also that the regions found to show positive correlation with FMA scores were only the regions showing lesser connectivity in CPH than PPH.

## Discussion

To the best of our knowledge, this is the first study to use resting-state functional connectivity analysis to investigate subgroups of stroke patients with different outcomes in hand function. The differences in functional connectivity in both the ipsilesional and contralesional M1s were assessed between each pair of the three diagnostic groups.

### Connectivity of the Ipsilesional M1 between Each Pair of the Three Diagnostic Groups

One previous study [33] reported reduced connectivity between the ipsilesional M1 and the contralesional hemispheric cortex after unilateral injury of the motor network. Connectivity of the ipsilesional M1 with contralesional M1 and postcentral gyrus significantly improved underlying motor recovery, and these connections significantly correlated with the degree of motor recovery [31]. In agreement with previous studies, we found significantly reduced connectivity of the ipsilesional M1 with contralesional cortical areas, such as contralesional middle frontal gyrus, superior frontal gyrus and cerebellum posterior lobe in PPH patients compared with the HC group. In contrast, we observed reduced connectivity of the ipsilesional M1 with contralesional S1M1 in CPH patients compared with the HC group. This finding suggests a breakdown of the normal interactions between ipsilesional M1 and the contralesional hemisphere, which may be associated with functional outcomes of the hand. In stroke patients with good functional recovery, regional activation had returned to normal. However, connections within the extended motor network (e.g., connection with prefrontal cortex) remained abnormal, and abnormal connectivity of the prefrontal cortex important implications for motor preparation and planning [37].

Studies in healthy volunteers have suggested that activity of the prefrontal cortex is more prominent during motor imagery in the absence of motor execution [38], [39]. In our study, stroke patients with PPH have regained certain practical abilities in hand function. Abnormal connectivity of the prefrontal cortex is likely a major determinant of deficits in motor preparation and planning rather than motor execution. In addition, our results suggest that outcomes in hand function might be determined by the preservation of connectivity between the ipsilesional M1 and contralesional S1M1. Disconnections of the ipsilesional M1 with the contralateral S1M1 are likely to result in CPH. Moreover, we found that the connectivity of the ipsilesional M1 with contralesional postcentral gyrus and superior parietal lobule significantly decreases in CPH compared with PPH, and they positively correlates with FMA scores (hand+wrist) across all stroke patients. It is well known that the higher FMA scores, the better motor function. Therefore, the positive correlations further suggest that the preservation of connectivity of the ipsilesional M1 with contralesional postcentral gyrus and superior parietal lobule implicate good recovery of hand function.

Our results also demonstrate significantly increased connectivity of the ipsilesional M1 with the ipsilesional cortical areas, such as the ipsilesional premotor cortex, prefrontal gyrus and parietal lobe in both the PPH and CPH patients compared with the HC group. This finding supports the idea that connectivity of ipsilesional M1 increased within ipsilesional brain regions, [33] which may reflect abnormalities of motor network interactions after a stroke, as well as plastic changes that compensate for the impaired connectivity with the contralesional hemisphere or the response to the disconnection of transcallosal inhibition.

Previous studies using task-based fMRI have demonstrated increased activations in the premotor cortex, prefrontal gyrus, parietal lobe and occipital lobe after a stroke [4], [7], [11]. Our findings also provide evidence of compensation in regional activity. In particular, we determined that connectivity of the ipsilesional M1 correlated positively with ipsilesional IPL. Early recruitment and high activation of the ipsilesional IPL was associated with faster or improved motor recovery [7] As suggested in a previous study, [4] the corticospinal tract appears to have the potential to be bypassed via alternative motor pathways. Instead of descending directly from the primary motor cortex, the corticospinal tract may project corticocortical connections to the SMA or premotor cortex. In addition, the IPL might be part of this alternative pathway [7] Indeed, the IPL was regarded as a site for both higher-order processing of sensory information and sensorimotor integration, which integrates perception and action [40].

### Connectivity of the Contralesional M1 between Each Pair of the Three Diagnostic Groups

We were also interested in exploring what patterns of connectivity of contralesional M1 may appear in subgroups of stroke patients with different outcomes in hand function compared with HC. Superficially, connectivity of contralesional M1, which mainly dominates the movement of the unaffected hand in stroke patients, may not differ from that in HC. In response to the above concern, the patterns of connectivity of contralesional M1 have also been revealed between each pair of the three diagnostic groups, which has rarely been analyzed in previous studies.

Compared with the HC group, PPH patients display reduced connectivity of the contralesional M1 with bilateral prefrontal gyrus and contralesional cerebellum posterior lobe. Instead, connectivity with the ipsilesional postcentral gyrus and bilateral cerebellum posterior lobe was reduced in the CPH group. The patterns of reduced connectivity in contralesional M1 are quite similar to the reduced connectivity of ipsilesional M1 in both the PPH and CPH groups compared with the controls, which suggests that the disruption of connectivity in contralesional M1 also occurs after stroke.

One previous study [41] reported reduced performance in bilateral movements in the unaffected hand in stroke patients, and other studies examining bimanual coordination in stroke patients also consistently reported this finding [42], [43]. The origin of a bimanual deficit after a stroke may be found in the reduction of promoting activity from the ipsilesional motor areas to contralesional M1 [41]. Our current results exhibit reduced connectivity of contralesional M1 in both the PPH and CPH groups, which may support the findings of previous studies. We speculate that reduced connectivity of contralesional M1 is likely to contribute to the deficit in bimanual coordination of bilateral movements rather than motor performance of the unilateral, unaffected hand. Furthermore, it is possible that disconnections in contralesional M1 with ipsilesional postcentral gyrus and bilateral cerebellum posterior lobe may play a crucial role in bimanual coordination of motor execution of bilateral movements and that the disconnection of contralesional M1 with bilateral prefrontal cortex may affect bimanual coordination of motor preparation or planning of bilateral movements.

An intriguing and unpredicted pattern of the connectivity of contralesional M1 has also been revealed. Indeed, there is increased connectivity of contralesional M1 with a wide range of motor-related areas, including bilateral precentral gyrus, bilateral SMA, ipsilesional cingulate gyrus and middle frontal gyrus, in CPH compared with HC. However, a smaller amount of connectivity of contralesional M1 was observed in the PPH group compared to controls. One study suggested that the role of the contralesional hemisphere is to provide ipsilateral motor pathways (also called uncrossed motor pathways) originating from the contralesional sensorimotor cortex. Though the ipsilateral motor pathways probable responding for small, it is a demonstrable portion of the total number of descending pathways in the brain [4]. The ipsilateral motor pathways have been detected in normal individuals via transcranial magnetic stimulation [44]. Moreover, ipsilateral responses were recorded at significantly lower thresholds after the stimulation of unaffected hemispheres in patients who had poor recovery after having a stroke, but this was not observed in patients with good recovery [45]. Consistent with previous studies, we conclude that the increased connectivity of contralesional M1 with a wide range of motor-related areas in CPH may contribute to an over-recruitment of ipsilateral motor pathways. In contrast, we speculate that the increased connectivity of contralesional M1 may be the consequence of over-use of the unaffected hand in CPH patients due to their need to substitute for the completely disabled hand for routine tasks.

### Limitations of our Study

Our study has several limitations. The first is the relatively small sample size of each group. Replicating our findings using a larger sample size of stroke patients is needed. In addition, the further subdivision of stroke patients with PPH may exhibit a more detailed relationship between the patterns of functional connectivity and outcomes in hand function. Second, we do not fully understand the dynamic changes in the patterns of functional connectivity following a stroke due to our cross-sectional design. Whether those patterns in cortical connectivity revealed by our current study can predict clinical outcomes during stroke recovery warrants longitudinal studies. Finally, we did not assess bimanual coordination in our stroke patients, which would have helped to understand the relationship between the connectivity of contralesional M1 and bimanual deficit after a stroke. We recommend that future studies perform this assessment.

## Conclusions

Our current study suggests, for the first time, that different outcomes in hand function in subgroups of stroke patients may be attributed to specific patterns of cortical connectivity. Therefore, reorganization of cortical connectivity may not only serve as a potential biomarker of neural substratum associated with hand function after subcortical stroke, but may also provide a valuable reference for understanding post-stroke outcomes of hand function. In addition, resting-state functional connectivity reflecting functional integrity could provide an effective method for evaluating the efficiency of post-stroke rehabilitative therapies.

## Supporting Information

Figure S1 **T2-weighted images show lesion (red arrow) of each stroke patient at the axial cross-section with the largest area.** Arabic numbers denote the case numbers of the stroke patients (PPH: partial paralyzed hands, CPH: completely paralyzed hands).
(TIF)

Figure S2
**Illustration of Paralyzed Hand Function Assessment: 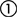 the affected hand stabilizes a piece of paper on the table, with the unaffected hand controlling a shear to cut the paper; 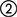 the affected hand holds a wallet, with the unaffected hand taking a coin from the wallet; 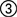 the affected hand holds an unfolded umbrella in the air for at least 10 seconds; 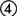 the affected hand controls a nail scissor to trim nails of the unaffected hand; and 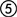 the affected hand buttons the cuff of the unaffected side.** The hand in dark denotes the affected hand.(TIF)
