# Supplementary figures and images for: Patterns in Cortical Connectivity for Determining Outcomes in Hand Function after Subcortical Stroke
Source: PLoS One. 2012 Dec 20;7(12):e52727. doi: 10.1371/journal.pone.0052727 (PMC3527607; doi:10.1371/journal.pone.0052727)

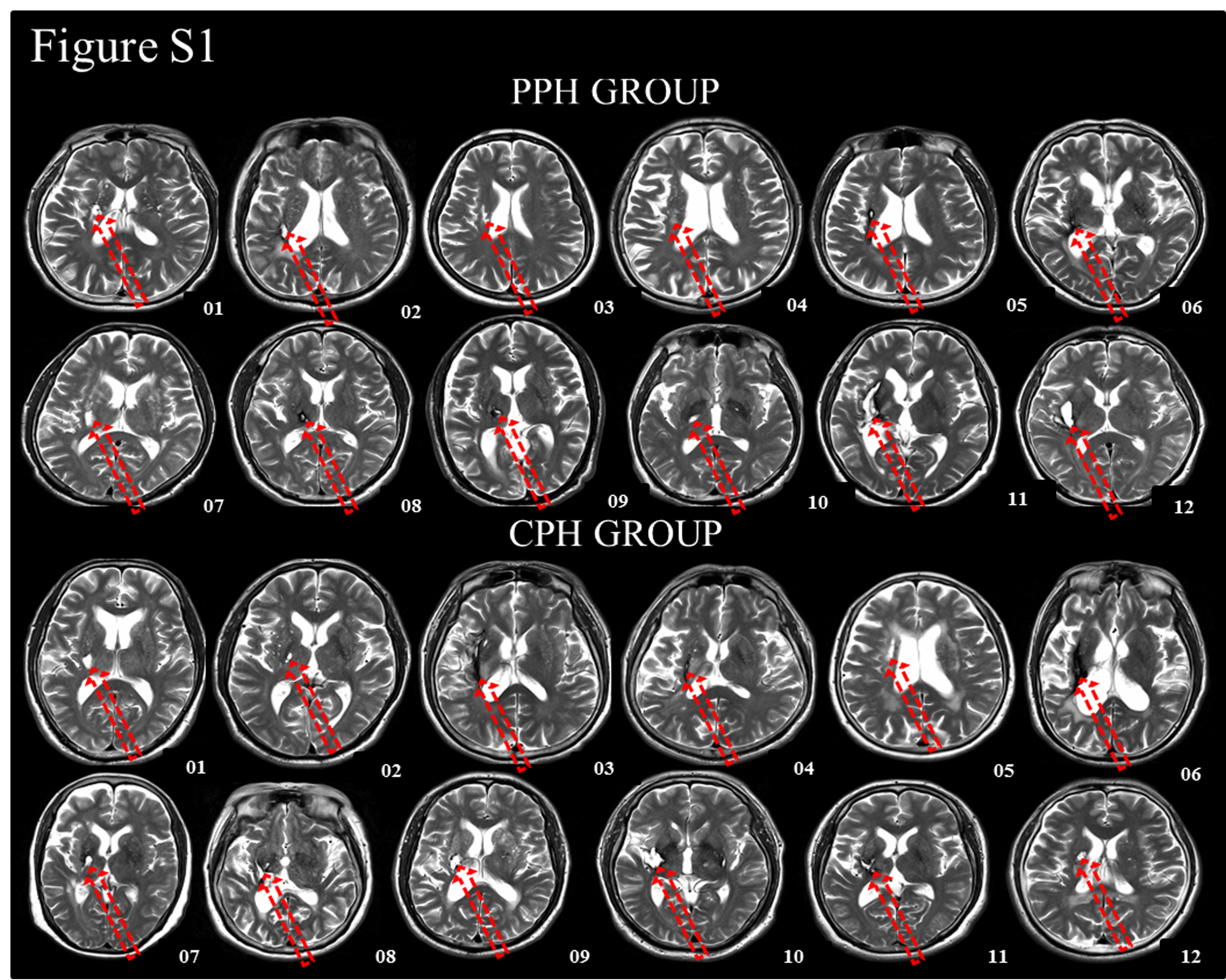

Supplement: Figure S1 — T2-weighted images show lesion (red arrow) of each stroke patient at the axial cross-section with the largest area. Arabic numbers denote the case numbers of the stroke patients (PPH: partial paralyzed hands, CPH: completely paralyzed hands). (TIF) [file pone.0052727.s001.tif]

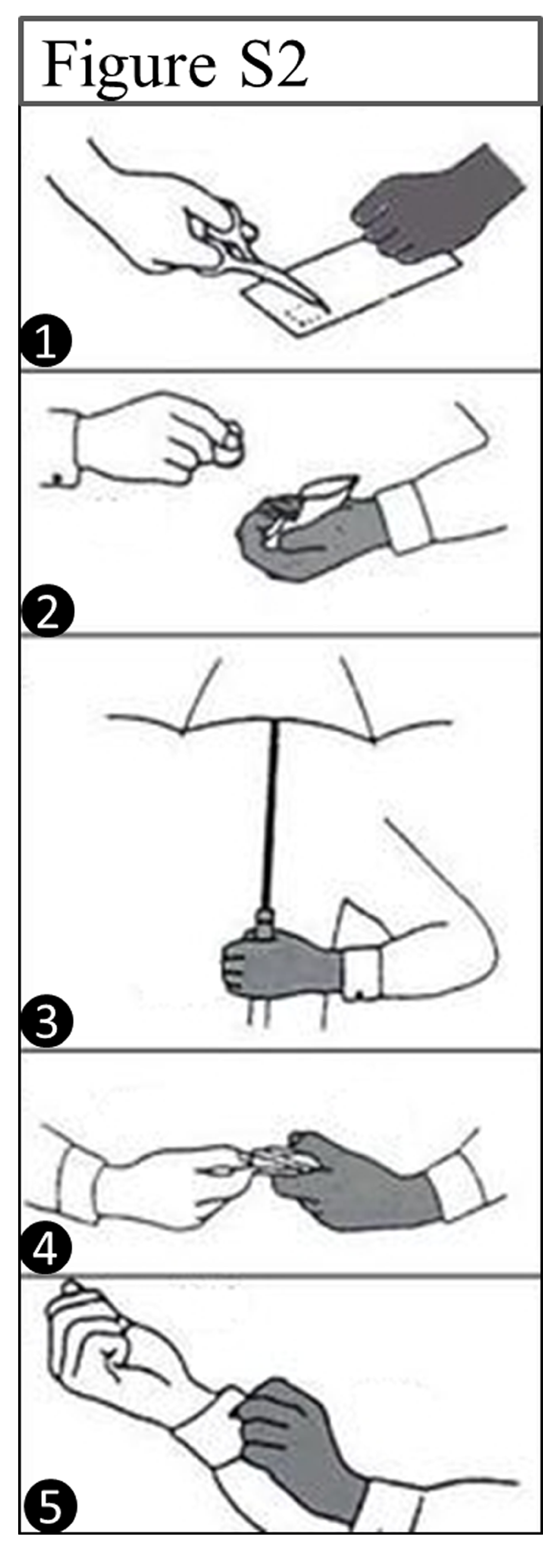

Supplement: Figure S2 — Illustration of Paralyzed Hand Function Assessment: the affected hand stabilizes a piece of paper on the table, with the unaffected hand controlling a shear to cut the paper; the affected hand holds a wallet, with the unaffected hand taking a coin from the wallet; the affected hand holds an unfolded umbrella in the air for at least 10 seconds; the affected hand controls a nail scissor to trim nails of the unaffected hand; and the affected hand buttons the cuff of the unaffected side. The hand in dark denotes the affected hand. (TIF) [file pone.0052727.s002.tif]
